# Elevation and persistence of CD8 T-cells in HIV infection: the Achilles heel in the ART era

**DOI:** 10.7448/IAS.19.1.20697

**Published:** 2016-03-03

**Authors:** Wei Cao, Vikram Mehraj, Daniel E Kaufmann, Taisheng Li, Jean-Pierre Routy

**Affiliations:** 1 Chronic Viral Illness Service, McGill University Health Centre, Montreal, QC, Canada; 2 Research Institute of the McGill University Health Centre, Montreal, QC, Canada; 3 Department of Infectious Diseases, Peking Union Medical College Hospital, Chinese Academy of Medical Sciences, Beijing, China; 4 Centre de recherche du Centre Hospitalier de l'Université de Montréal, Montreal, QC, Canada; 5 Department of Medicine, Université de Montréal, Montreal, QC, Canada; 6 Division of Hematology, McGill University Health Centre, Montreal, QC, Canada

**Keywords:** HIV, antiretroviral therapy, CD8 T-cell persistence, bystander activation, T-cell exhaustion

## Abstract

**Introduction:**

HIV infection leads to a disturbed T-cell homeostasis, featured by a depletion of CD4 T-cells and a persistent elevation of CD8 T-cells over disease progression. Most effort of managing HIV infection has been focused on CD4 T-cell recovery, while changes in the CD8 compartment were relatively underappreciated in the past.

**Methods:**

A comprehensive literature review of publications in English language was conducted using major electronic databases. Our search was focused on factors contributing to CD8 T-cell dynamics in HIV infection and following antiretroviral therapy (ART).

**Discussion:**

Normalization of CD8 counts is seldom observed even with optimal CD4 recovery following long-term treatment. Initiation of ART in primary HIV infection leads to enhanced normalization of CD8 count compared with long-term ART initiated in chronic infection. Importantly, such CD8 elevation in treated HIV infection is associated with an increased risk of inflammatory non-AIDS-related clinical events independent of CD4 T-cell recovery. The mechanisms underlying CD8 persistence remain largely unknown, which may include bystander activation, exhaustion and immunosenescence of CD8 T-cells. The information provided herein will lead to a better understanding of factors associated with CD8 persistence and contribute to the development of strategies aiming at CD8 normalization.

**Conclusions:**

Persistence of CD8 T-cell elevation in treated HIV-infected patients is associated with an increased risk of non-AIDS-related events. Now that advances in ART have led to decreased AIDS-related opportunistic diseases, more attention has been focused on reducing non-AIDS events and normalizing persistent CD8 T-cell elevation.

## Introduction

Human immunodeficiency virus (HIV) infection is characterized by a profound immune dysfunction and a disturbed T-cell homeostasis. While progression of the infection is associated with a gradual loss of CD4 T-cells, the CD8 T-cell count (hereinafter referred to as CD8 count) is elevated at the very onset of infection and during the chronic phase until the late phase where major depletion of all T-cell subsets occurs.

For a fairly long time, management of HIV infection has been primarily based on CD4 T-cell recovery, while changes in the CD8 compartment were relatively neglected. Due to a significant CD4 T-cell recovery with antiretroviral therapy (ART) over the last decade, studies began to assess the dynamics of CD8 count and its prognostic significance in treated HIV infection. Although effective ART has greatly reduced AIDS-related events in the majority of HIV-infected patients, normalization of CD8 counts is seldom observed even with optimal CD4 recovery [1,2]. Moreover, such elevation of CD8 counts is associated with an increased risk of non-AIDS-related clinical events, including malignancies and cardiovascular diseases, independent of CD4 T-cell recovery [1,3].

CD8 T-cell population is particularly disturbed during HIV infection, leading to a unique persistent elevation and dysfunction of CD8 T-cells. This elevation, not fully restored by effective ART, may represent the remaining Achilles heel in the current era of HIV care as it has been linked with risk of non-AIDS-related events. In this review, we will discuss the shifted dynamics of CD8 T-cells during HIV infection and the underlying mechanisms. The information provided herein will contribute to a better understanding of CD8 persistence under ART-induced viral suppression and pave the way for further strategies towards CD8 T-cell normalization.

## Methods

A comprehensive review of the English-language publications was conducted. We searched Ovid Medline, JSTOR and Scopus electronic databases with keyword combinations including CD8 T-cell count(s), CD8 T-cell persistence/elevation, CD4/CD8 ratio, immune activation, bystander activation, T-cell exhaustion and immunosenescence. The same strategy was used with Google Scholar and ISI Proceedings to further include non-peer-reviewed literature and conference publications. For describing immune changes during HIV infection and other viral infections, we included studies from HIV-1-infected humans, simian immunodeficiency virus (SIV)-infected monkey models and lymphocytic-choriomeningitis-virus (LCMV)-infected murine models. For information related to treatment effect on immune cells, our search was limited to HIV-1-positive adults using publications from 2000 to 2015 at a time when advances in ART have allowed long-term control of viral replication.

## Results and discussion

### CD8 T-cell elevation: lessons from cytomegalovirus infection and ageing

The *in vivo* dynamics of human CD8 count and its subsets are influenced by several intrinsic and extrinsic factors, such as age, gender, physical activity, smoking, alcohol consumption and comorbidity including chronic viral infections [4–6]. Among these factors, the effects of ageing and cytomegalovirus (CMV) infection have been most extensively studied.

Ageing is associated with an increase in the circulating CD8 T-cells and an expansion in memory and late-stage T-cell subsets, predominantly in the CD8 rather than CD4 compartment [7,8]. These accumulated late-stage memory CD8 T-cells are characterized by decreased expression of the “functional fitness” marker CD28, an important co-stimulatory receptor, and enhanced expression of the carbohydrate CD57, thus designated as the “immunosenescence” marker. In many individuals, a significant fraction of these senescent CD8 T-cells is directed towards CMV, whose prevalence increases with ageing and accumulative antigen exposure [7]. By 1990s, an immune risk phenotype (IRP) has been developed in non-HIV-infected elderly people (>85 years old) to define a phenotype characterized by CMV IgG sero-positivity, a low CD4/CD8 ratio mainly due to the accumulation of CD8 T-cells and an abnormally high frequency of circulating CD28^neg^ T-cells [9,10]. As demonstrated by many studies, IRP represents a marker of biological ageing of the immune system and has been validated to be independently associated with morbidity and mortality in the elderly [7,8,11,12].

Similar to the immune alterations observed in IRP, HIV-infected patients also present with low CD4/CD8 ratio, elevated CD8 counts and an expansion of the memory CD8 T-cell subsets [13]. It was recently reported that despite effective ART, HIV-infected patients with elevated IRP displayed a higher degree of immune senescence than their non-IRP counterparts [14]. The significant overlap in clinical and immunological phenotypes observed during normal ageing and HIV infection has raised the concept of premature senescence in HIV infection. All these contributors, intermingled with prolonged life expectancy, have renewed the interest in CD8 T-cell elevation in HIV infection.

### The unremitting elevation of circulating CD8 T-cells in treated HIV infection

Elevation and expansion of CD8 T-cells occurs from the very early days of HIV infection, as observed in other acute viral infections. During this phase, the rapid and robust expansion of CD8 T-cells particularly in the viral-specific subsets contributes to a partial control of viraemia [15,16]. It has also been demonstrated in SIV-infected non-human primates that an early increase in CD8 T-cells following therapy suspension was associated with a subsequently lower viral load [17]. However, unlike other viral infections where elevation of CD8 T-cells subsides with the clearance of antigen, the expansion and elevation of CD8 T-cells persists throughout HIV infection. Over time, the terminally differentiated CD8 subsets are dramatically elevated, while the naïve and central memory CD8 T-cells progressively declined [18–20].

Although effective ART could achieve a viral control and CD4 T-cell recovery in the majority of patients, quantitative and functional defects in CD8 T-cells remain even after a decade of treatment [2,21]. Following a modest decrease after ART initiation, the CD8 counts remain consistently elevated and relatively stable over time [1,3,22]. In these long-term treated patients, we assessed factors that were associated with CD8 T-cell elevation [23]. Younger patient age and the female gender were associated with lower CD8 counts, while duration of ART was not even after more than one decade. Although the turnover of CD8 T-cell subsets was partially recovered following long-term treatment, higher-than-usual levels were still observed in all the CD8 T-cell subsets, especially in the memory and activated subsets [21,24]. Higher frequency of naïve CD8 T-cells was associated with lower CD8 count and higher CD4/CD8 ratio in long-term treated patients, indicating that changes in the memory subsets are the major drive for the overall CD8 T-cell elevation [25].

**Figure 1:**
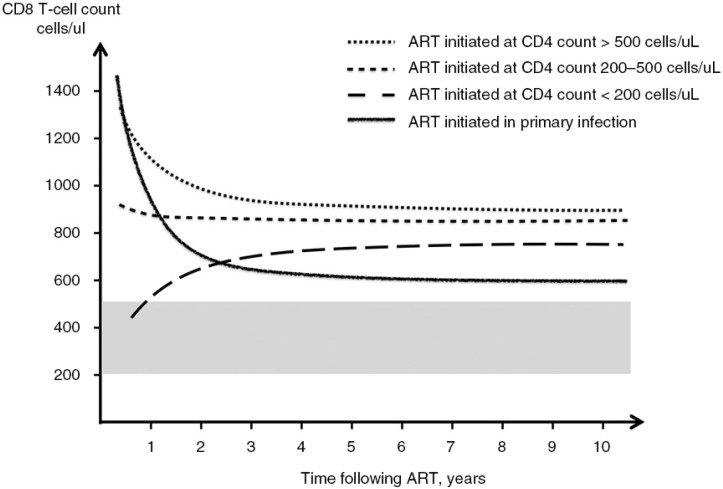
**CD8 T-cell dynamics following ART initiation in HIV infection**. Changes of CD8 counts after ART initiation in primary HIV infection and chronic HIV infection according to pre-ART CD4 counts. Reference range from the uninfected population was shown in grey shade. Early initiation of ART results in a lower level of CD8 counts compared with delayed therapy regardless of baseline CD4 count and remains stable over time. Figure modified from [1] and [22].

Changes of CD8 count in primary HIV infection and following early initiated ART have been less well studied. Recently, we reported that ART initiated in primary infection was associated with a CD8 count decrease to a level lower than that in long-term treated chronic patients and remained stable over time [22]. Early timing of ART initiation had privileged trend to CD8 count normalization compared with ART initiated in chronic infection, highlighting that shorter duration of antigen exposure is associated with both reduced immune activation and lower CD8 elevation [22,26] (Figure 1).

Interestingly, such CD8 persistence not only takes place in the periphery but also in multiple lymphoid tissues. A significant expansion of CD8 T-cells enriched in the memory subsets was observed in lymph nodes of HIV-infected patients [27,28]. Recently, a study reported that an early accumulation of CD8 T-cells in the duodenal mucosal associated lymphoid tissue occurs in parallel with the periphery during primary HIV infection. Interestingly, the CD8 T-cell accumulation was partially resolved following early ART initiation [29]. These changes echoing in the periphery and lymphoid tissues indicate that the elevation of CD8 count is more than a shifted compartment distribution.

Persistently elevated CD8 counts following long-term ART have been linked with increased risks of non-AIDS-related events in HIV-infected patients, independent of CD4 T-cell recovery [1,3,30]. These include but are not limited to cardiovascular, renal, respiratory, metabolic diseases and non-AIDS-defining malignancies, which have been independently linked to status of chronic inflammation, level of microbial translocation and immune activation markers such as IL-6, soluble CD14 and d-dimer [3,31–33].

### Mechanism of CD8 T-cell elevation: a “ménage-a-trois” of immune activation, exhaustion and immunosenescence

#### Bystander CD8 T-cell activation: more than just standing by

Unlike most other viral infections, HIV leads to a marked and durable activation not only in HIV-specific but also in non-specific CD8 T-cells, referred to as “bystander” activation [34]. HIV-specific CD8 T-cells represent between 8% and 50% of total circulating CD8 T-cells, which further decrease following acute infection and/or ART initiation [35–37]. The bystander expansion is induced by T-cell-receptor-independent mechanisms mainly including cytokine and chemokine stimulation [38,39]. CD8 T-cells are expanded and activated regardless of antigen specificity, including subsets specific to persistent and non-persistent antigens such as CMV, Epstein–Barr virus (EBV), influenza virus and adenovirus [34,40]. The mechanisms underlying such bystander activation remain unclear. However, the broader and less restrictive mode of expansion in CD8 T-cells compared with that of the CD4 compartment suggested that cytokine stimulation and inflammatory environment play an essential role in bystander CD8 expansion.

Studies in mice infected with LCMV showed that bystander T-cells, especially the memory subsets, generally underwent attrition and apoptosis, making space for the upcoming antigen-specific T-cells [41]. However, there was also evidence that pre-existing memory CD8 T-cells specific for previous encountered infections were largely preserved when long-lived memory CD8 T-cells specific to a new vaccine antigen were developed [42]. The fate of bystander CD8 T-cells in HIV infection remains ill defined. Interestingly, both bystander CD8 activation in HIV infection and bystander CD8 apoptosis in LCMV infection were observed with a peak of several cytokines including type-I interferon (IFN) such as IFN-α/β, providing some insight into mechanisms regulating the bystander CD8 T-cell population [40,41]. Recent studies also showed that auranofin, a gold-based compound, could cause apoptosis and renovation in both CD4 and CD8 memory T-cells and therefore led to a reduction in the post-therapy viral set point in treated SIV infection [17,43]. Some also indicated that the dysfunction of the CD4 regulatory T (Treg) cells due to HIV infection led to a failure to control the bystander activation and contributed to the overexpansion of CD8 T-cells [44–46].

#### Elevated CD8 T-cells: controlled at checkpoints

**Figure 2:**
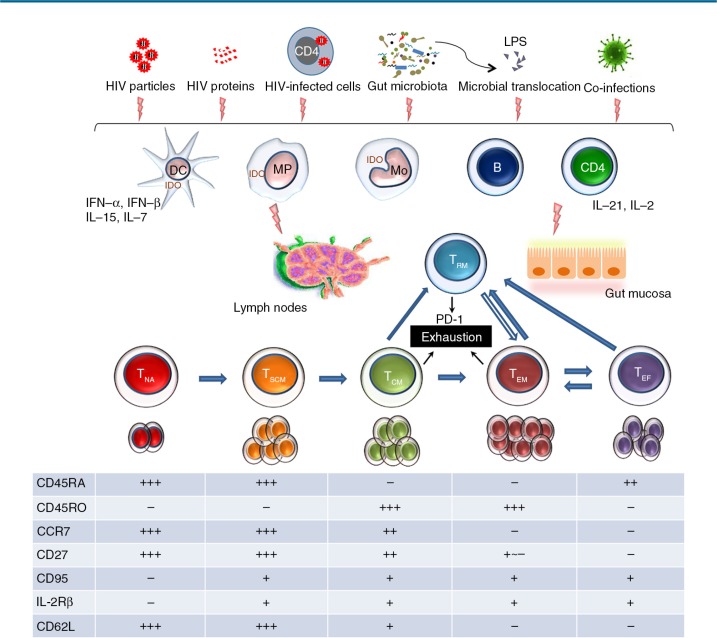
**CD8 T-cell exhaustion during chronic HIV infection**. In addition to viral factors including HIV particles, proteins and infected cells, long-lasting gut microbiota changes and microbial translocation induced by HIV also act as stimulant for the immune system during chronic infection. Both the innate and the acquired immune systems are involved in the process of CD8 T-cell proliferation, differentiation and exhaustion. Characteristic expression of surface markers on each CD8 T-cell subset is shown in the table. The majority of exhausted CD8 T-cells originate from memory subsets including T_CM_, T_EM_ and T_RM_, which is the major source of exhausted CD8 T-cells [59–61]. DC, dendritic cell; IFN, interferon; IL, interleukin; LPS, lipopolysaccharide; MP, macrophage; Mo, monocyte; T_CM_, central memory CD8 T-cell; T_EF_, effector CD8 T-cell; T_EM_, effector memory CD8 T-cell; T_NA_, naïve CD8 T-cell; T_RM_, tissue-resident memory CD8 T-cell; T_SCM_, stem central memory CD8 T-cell.

In addition to the extensive expansion and activation of memory CD8 T-cells, a dysregulated turnover of these CD8 T-cells involving exhaustion and senescence further contributes to the CD8 accumulation over disease progression [47]. Such T-cell exhaustion starts soon after peak viremia and lasts throughout the course of infection, reflecting a compromise between the host and the invading virus [48] (Figure 2). Features of CD8 T-cell exhaustion include reduced proliferative potential and cytokine production, loss of the central memory phenotype and acquired co-expression of multiple inhibitory receptors such as programmed cell death protein-1 (PD-1), lymphocyte activation gene-3 protein (LAG-3), CD160 and T-cell immunoglobulin mucin-3 (Tim-3) [49–51]. Chronic and persistent exposure to the viral antigen, lack of CD4 T-cell help and sustained co-expression of multiple inhibitory receptors characterize CD8 T-cell exhaustion in HIV infection. Although CD4 and CD8 T-cells share many features of exhaustion, CD8 T-cells have their distinct set of inhibitory receptors including a preferential expression of CD160 and 2B4 and more enhanced expression of PD-1 [52] (Table 1). Recent findings suggested that the retention of a demethylated PD-1 locus in HIV-specific CD8 T-cells of ART-treated patients and elite controllers resulted from epigenetic programming acquired by prolonged antigenic exposure [52,53]. This epigenetic change of the PD-1 transcriptional regulatory region persists for years after suppression of viral load and poises the PD-1 gene for higher expression in virus-specific CD8 T-cells. More importantly, dysfunctional CD4 T-cells also contribute to the expansion and exhaustion of the CD8 compartment, since impaired production of IL-21 from CD4 T-cells leads to a substantial CD8 T-cell dysfunction as demonstrated in chronic LCMV infection and HIV infection [54–57]. In the murine model, administration of IL-21 has been shown to lead to preferential expansion of naïve CD8 T-cell subsets, which may contribute to normalization of CD8 T-cell homeostasis in the long run [58–61].

**Table 1:** Features of CD8 and CD4 T-cell exhaustion in HIV infection

Features	CD8 T-cells	CD4 T-cells
Antigen-persistence driven	+	+
Proliferative potential and self-renewal	Low	Low
Cytokine production	↓ TNF, IFN-γ, IL-2	↓ TNF, IFN-γ, IL-2, IL-21
		↑ IL-10
Lineage skewing	Skewed to late memory subset	Th1→Tfh-like phenotype
Inhibitory markers	↑ PD-1, LAG-3, CD160, Tim-3, CD244 (2B4)	↑ CTLA-4, PD-1, Tim-3
Reversible with ART	+/–	+ +

Tfh, T follicular helper cells; PD-1, programmed cell death protein 1; LAG-3, lymphocyte activation gene 3 protein; CTLA-4, cytotoxic T-lymphocyte antigen 4; Tim-3, T-cell immunoglobulin mucin-3.

In contrast to the well-recognized role of T-cell exhaustion, the concept of CD8 T-cell senescence induced by HIV remains controversial. Immunosenescence is characterized by the accumulation of late-differentiated memory CD8 T-cells harbouring low replicative capacity. These CD8 T-cells are characterized by loss of the co-stimulatory molecule CD28 with enhanced expression of CD57 and harbouring shortened telomeres [62,63]. Compared with the age-matched uninfected controls, HIV-infected patients presented with increased frequencies of CD28-CD57 + CD8+ T-cells, which tended to have poor proliferative capacity and were specific to several other viral antigens.

However, the role of HIV in immunosenescence remains controversial, as most of the detected senescent CD8 T-cells are CMV-specific, which are already identified *per se* as a risk factor of immunosenescence [64]. In addition, the expanded CMV-specific T-cells possess rather similar phenotypic and functional features to those of replicative senescent T-cells [65,66]. Recently, a study examined the senescent CD8 T-cell phenotypes in HIV patients with asymptomatic CMV infection and in non-HIV-infected adults with or without CMV infection [67]. It was found that unlike CMV and ageing, HIV inhibited the process of terminal differentiation, expanded the less-differentiated transitional memory and CD28-CD57-CD8+ T-cells, therefore decreasing the proportion of CD28-CD57 + CD8+ T-cells [67]. Of note, such low proportion of CD28-CD57 + CD8+ T-cells was also an independent predictor for increased mortality in treated HIV infection [68]. These observations are consistent with previous studies indicating chronic HIV infection drives HIV-specific CD8 T-cells towards a moderately differentiated phenotype (CD28-/CD27 + ), in contrast to the more differentiated (CD28-/CD27-) phenotype in CMV-specific CD8 T-cells [69]. Therefore, it is possible that HIV contributes to the expansion and exhaustion of memory CD8 T-cells, with a more prominent proportion of CMV-specific compartments skewed towards replicative senescence.

### Determinants of CD8 T-cell persistence under conditions of low antigenic stimulation during treated HIV infection

The mechanisms underlying persistent CD8 T-cell elevation in treated HIV infection remain largely unidentified. Based on previous reports in treated chronic HIV infection and our findings in primary infection, we speculate that CD8 elevation may contain two compartments, a fast responsive and a persistent one. The fast-responsive compartment most probably includes activated CD8 T-cells (CD38 + /HLA-DR + ) driven by viral antigens and can be restored following early ART initiation [26]. The persistent one may be maintained by residual viral exposure including HIV particles and proteins, gut mucosal dysfunction and microbial translocation. Multiple humoral and cellular players from both the innate and acquired immune systems contribute to the persistence of CD8 T-cells; yet their relative contribution remains unclear and their causal effect has to be determined (Figure 2).

#### An altered proinflammatory and anti-inflammatory cytokine milieu

HIV infection generates a systemic inflammatory environment and shifted profiles of inflammatory cytokines. As both proliferation and differentiation of CD8 T-cells are predominantly driven by HIV replication along with inflammatory environment, alterations of the cytokine milieu play a major role on CD8 T-cell homeostasis [70,71].

As one of the major sources causing chronic inflammation, the gut mucosal damage starts since early infection, leading to a modified microbiota and translocated microbial products, which is not reversed with early initiation of ART [26]. Plasma levels of microbial products remain elevated in long-term treated patients, which activate specific pattern receptors on APCs and induce stepwise proinflammatory cytokine production. Lipopolysaccharide (LPS) recognized by the Toll-like receptors (TLRs) on dendritic cells (DCs) enhances the production of type-I IFNs including IFN-α/β [72]. The accumulation of type-I IFNs further induces production of INF-γand interleukins (ILs) such as IL-15 and IL-7, which directly promote CD8 T-cell expansion. In addition, the type-I-IFN-dependent production of indoleamine 2,3-dioxygenase (IDO) was also detected in the lymphoid tissue of HIV-infected individuals, which catalyzes tryptophan degradation, further fuelling the gut mucosal damage, systemic inflammation and T-cell dysfunction [26,73].

Effects of IL-15 and IL-7 on CD8 T-cell elevation have been demonstrated by *ex vivo* observation that both cytokines triggered the proliferation and activation of memory CD8 T-cells, much more than CD4 T-cells, in a TCR-independent way [38,40,74]. In viral-suppressed SIV-infected non-human primates, this effect may further translate into a failure of CD4 T-cell reconstitution [75]. Increased IL-15 level was also observed in lymph node culture of untreated HIV-infected patients, where CD8 T-cells were disproportionally enriched in contrast to the depletion of CD4 T-cells [28]. Furthermore, increased tissue levels of IL-1β have been observed in both untreated and treated patients, which may also contribute to the expansion of memory CD8 T-cells [76]. On the other hand, inhibitory cytokines such as IL-10 and transforming growth factor-β (TGF-β) contribute to restoration of memory CD8 T-cell development via inhibition of the maturation and function of DCs and macrophages [77,78].

In addition, the plasma markers and phenotypes of monocyte activation were shown to be associated with increased inflammatory biomarkers such as IL-6 and hsCRP in viral-suppressed individuals [79]. The similar biomarker profile associated with CD8 elevation indicates a link between monocyte activation and CD8 persistence that has yet to be studied. Recently, we also demonstrated that extracellular vesicles including exosomes and microvesicles released by several cell types may also contribute to immune dysfunction and elevation of CD8 counts [80].

#### Distribution and trafficking of CD8 T-cells: does location matter?

The alteration of T-cell distribution is informative in assessing CD8 dynamics, since the circulating CD8 count is used in clinical practice. Such elevation may be partially due to failure of CD8 T-cell trafficking to lymphoid tissues. However, recruitment and migration of CD8 T-cells from circulation to sites of infection is an under-explored area in HIV infection. Results from SIV-infected rhesus macaques showed a significant loss of CD4 T-cells and activation of CD8 T-cells in the peripheral blood and lymph nodes a few weeks after infection, accompanied by an alteration of cytokine/chemokine profiles [81]. In contrast, despite a similar CD4 T-cell depletion, no evidence of CD4 T-cell exhaustion or CD8 T-cell activation in the bone marrow was detected, suggesting tissue-specific T-cell changes during acute SIV infection.

Similarly, in untreated HIV-infected patients, a progressive depletion of CD4 T-cells and a prominent expansion of CD8 T-cells have been demonstrated both in the peripheral blood and in lymphoid tissues [27,29]. Similar composition of the CD8 T-cell subsets in both blood and lymph nodes compared with that of the CD4 T-cell subsets suggests an expansion of CD8 T-cells in both compartments [27]. Moreover, these CD8 T-cells display an altered expression of adhesion molecules, leading to a higher adhesive capacity facilitating trafficking and persistence into the lymphoid tissue [27]. Immaturity and functional defects in these tissue-resident CD8 T-cells, which likely contributed to viral persistence, were also observed [82]. All the evidence indicates that peripheral CD8 count elevation owes little to the decreased trafficking of these cells to tissues. In contrast, the enhanced adhesion and trafficking of CD8 T-cells in HIV infection further promotes persistence of CD8 T-cells in lymphoid tissues, which may in turn serve as a source of peripheral CD8 persistence.

#### Co-morbidities: other uninvited guests

The co-existing chronic and persistent infections other than HIV may result in an expansion and exhaustion of CD8 T-cells. The level and duration of the chronic antigenic stimulation are key determinants for T-cell dysfunction. As stated above, CMV co-infection induces CD8 T-cell senescence, which is further aggravated by HIV infection [69]. Previous studies also showed that untreated HCV co-infection would hamper CD8 T-cell down-regulation in HCV/HIV co-infected individuals, despite sustained HIV suppression and CD4 T-cell recovery with ART [83,84]. As the immune dysfunction continues even in long-term treated patients with optimal CD4 T-cell recovery, it is possible that the immune system is frequently challenged and stimulated, which may contribute to chronic immune activation.

In addition, risk factors having been shown to play a role in the proinflammatory status in non-HIV individuals are also considered involved in non-AIDS events occurring in HIV-infected individuals, including hypercholesterolemia, hypertension, diabetes and smoking. The relative contribution of lifestyle risk factors and their downstream cascade on CD8 T-cell elevation remains to be explored.

### Potential strategies to intervene on CD8 T-cell elevation

While long-term ART results in moderate decrease in CD8 T-cell elevation, early initiated therapy reduces antigenic stimulation partially contributing to normalization of CD8 T-cell counts [22]. Several other strategies have been tested, which may contribute to a better control of HIV-related immune activation and CD8 persistence.

#### Reversal of CD8 T-cell exhaustion

Inhibitory receptors such as PD-1, CTLA-4, TIM-3, CD160, 2B4 and LAG-3 play a critical role in the maintenance of CD8 T-cell exhaustion. Blockade of such receptors alone or in combination represents a most promising therapeutic approach and has shown efficacy in reversing T-cell exhaustion *in vitro* and in animal models, and more encouragingly in cancer immunotherapy [85,86]. Therapeutic blockade of inhibitory pathways may enhance the function of cytotoxic CD8 T-cells with the ultimate objective to clear viral reservoirs. Other strategies include TLR-2 agonists, which reverse CD8 T-cell exhaustion and enhance pathogen-specific T-cell responses *in vivo*. Agonistic antibodies against 4-1BB or CD40 also seem to be promising [87].

#### Enhancing cytotoxic function by recombinant cytokines

Recombinant cytokines alone or in combination with inhibitory receptors can improve CD8 T-cell cytotoxicity and contribute to their tissue-resident function at the site of HIV persistence. Recombinant IL-15 enhances CD8 T-cell function as reported in animal models where the superagonist IL-15 (ALT-803) is being evaluated in a clinical trial with ART-treated HIV-infected subjects (ClinicalTrials.gov identifier: NCT02191098) [88,89]. The transient elevation of CD8 T-cells following IL-15 administration may lead to their post-treatment decay due to a reduction in HIV-associated inflammation.

#### Homing and/or trafficking strategy

As follicular CD4 T-cells in the germinal centres represent an important site of HIV persistence related to the exclusion of cytotoxic CD8 T-cell homing, strategies that may either disrupt germinal centre or modify CD8 T-cell homing in this site may contribute to a decrease in HIV persistence and in turn lower CD8 elevation [90,91].

Globally, these therapeutic strategies should encompass early ART initiation paired with immunotherapies and may contribute to achieve significant and durable change in T-cell function and homeostasis [92].

## Conclusions

CD8 T-cell persistence in blood and tissues is mainly a reflection of chronic inflammation and immune activation, where irreversible epigenetic changes occurred in these exhausted cells. In addition to the residual viral replication, persistence of viral proteins, gut mucosal damage and co-existing factors such as CMV infection also contributes to the CD8 persistence. Multiple players in the immune system are involved in this process, particularly the cytokine and chemokine network has been demonstrated to play a critical role. CD8 T-cell persistence remains the Achilles heel in the ART era representing underlying immunopathogenesis, while the functional skewing accompanying such quantitative CD8 elevation probably further fuels the immune dysfunction. Combination of innovative immunological strategies in addition to ART is required to enhance CD8 T-cell normalization and improve the life of persons living with HIV.
